# Determinants of preventive oral health behaviour among senior dental students in Nigeria

**DOI:** 10.1186/1472-6831-13-28

**Published:** 2013-06-18

**Authors:** Morenike O Folayan, Mohammad R Khami, Nkiru Folaranmi, Bamidele O Popoola, Oyinkan O Sofola, Taofeek O Ligali, Ayodeji O Esan, Omolola O Orenuga

**Affiliations:** 1 Department of Child Dental Health, Obafemi Awolowo University, Ile-Ife, Nigeria; 2 Dental Research Center, Community Oral Health Department, School of Dentistry, Tehran University of Medical Sciences, Tehran, Iran; 3 Department of Child Dental Health, University of Nigeria, Enugu, Nigeria; 4 Department of Child Oral Health, University of Ibadan, Ibadan, Nigeria; 5 Department of Preventive Dentistry, University of Lagos, Lagos, Nigeria; 6 Department of Preventive Dentistry, University of Maiduguri, Maiduguri, Nigeria; 7 Obafemi Awolowo University Teaching Hospitals Complex, Ile-Ife, Nigeria; 8 Department of Child Dental Health, University of Lagos, Lagos, Nigeria

**Keywords:** Nigeria, Dental, Students, Knowledge, Behaviour, Prevention

## Abstract

**Background:**

To study the association between oral health behaviour of senior dental students in Nigeria and their gender, age, knowledge of preventive care, and attitudes towards preventive dentistry.

**Methods:**

Questionnaires were administered to 179 senior dental students in the six dental schools in Nigeria. The questionnaire obtained information on age, gender, oral self-care, knowledge of preventive dental care and attitudes towards preventive dentistry. Attending a dental clinic for check-up by a dentist or a classmate within the last year was defined as preventive care use. Students who performed oral self-care and attended dental clinic for check-ups were noted to have complied with recommended oral self-care. Chi-square test and binary logistic regression models were used for statistical analyses.

**Results:**

More male respondents agreed that the use of fluoride toothpaste was more important than the tooth brushing technique for caries prevention (P < 0.001). While the use of dental floss was very low (7.3%), more females were more likely to report using dental floss (p=0.03). Older students were also more likely to comply with recommended oral self-care (p<0.001). In binary regression models, respondents who were younger (p=0.04) and those with higher knowledge of preventive dental care (p=0.008) were more likely to consume sugary snacks less than once a day.

**Conclusion:**

Gender differences in the awareness of the superiority of using fluoridated toothpaste over brushing in caries prevention; and in the use of dental floss were observed. While older students were more likely to comply with recommended oral self-care measures, younger students with good knowledge of preventive dental care were more likely to consume sugary snacks less than once a day.

## Background

Oral self-care practice is an effective preventive measure for maintaining good individual oral health which is an integral part of one’s general health. For dental health professionals, their health beliefs and attitudes not only affect their oral self-care habits but may also potentially influence their ability to motivate patients to undertake preventive oral health measures [1,2]. This in turn has an impact on the public’s understanding of preventive oral health measures [3]. It is in view of this important link that Kawamura et al [4], advocated for undergraduate dental education to include comprehensive programmes in preventive care that empowers dentists to motivate patients for oral self-care in addition to programmes that ensure dental students also institute oral self-care regimens. Such educational effort should enable dental students develop stable health behaviours [2] which are not influences by individual characteristics [5,6].

The relationship between knowledge, attitude and practices has been previously demonstrated [7]: knowledge, attitude, understanding and competency are predispositions to act. The relation between knowledge, attitude and practice seems to be stronger among professionals compared to lay people [3,5].

The place and importance of background factors that may inform socialization processes cannot be overlooked. For example, gender differences observed in the frequency of daily tooth brushing in the community was not observed among professional dentists in Mongolia [5]. Similarly, gender differences in the tooth brushing frequency among dental professionals was not observed in Australia, China, Finland, Hong Kong, Japan, Korean and the US [8-10] This has been explained as the role of professional education in overcoming such differences [5,11]. Gender differences in tooth brushing practices were however, still observed among Greek and Jordan dental students despite exposure to professional education [6,12].

There are also differing reports on the possible impact of professional education on frequency of sugar consumption: in Mongolian the students’ frequency of sugar-consumption remained unchanged despite their professional education [5] while in Finland, professional education did make a difference in the frequency of sugar consumption by dental students [13].

The need to promote the practice of preventive dental care in Nigeria is critical because the available resources for health care in Nigeria as in most developing countries are inadequate to support the traditional curative care of dental diseases [14]. It is therefore important to study and understand how much value the final year dental students place on prevention. Such value will be reflected in the adoption of preventive oral health care practices for themselves.

The present study therefore investigated the association between the oral health behaviour (oral self-care and preventive care use) of senior dental students in Nigeria with their age, gender, knowledge of caries prevention, and attitudes towards preventive dentistry.

## Methods

A self-assessment questionnaire was used as a survey instrument for this study. Khami et al’s methodology was adopted for this study [15]. The questionnaire used in the study conducted by Khami et al. [15], was pilot tested amongst five dental students who finished dental school within two months of piloting the questionnaire. Specific details on the questionnaire were adjusted based on outcomes of the discussions held with the students. Revision of the questionnaire was based on ease of understanding and interpretation of contents of the questionnaire during the pilot study. The questionnaire was also adjusted in view of the study objectives and the context of the practice of dentistry in Nigeria. For example, the Iranian questionnaire explore for the role of past training as a dental hygienist in preventive practice. This questionnaire did not include those set of questions that explored for the role of past training as a dental hygienists in preventive practice since there is no such history of dental hygienist becoming trained as dentist in the dental education history of Nigeria. Secondly, the questions about basic qualifications were revised to reflect the types of possible qualifications that could be obtained in Nigeria. Thirdly, the questionnaire asked about working experience. Since the questionnniare was administered to students, this question was excluded.

Estimated sample size for assessment of preventive oral health behaviour of dental students using a prevalence of 47% reported by Khami et al [15], 95% confidence interval and an error margin of 5% was 382.

The target population of this study comprised of the final year dental students in six of the eight dental schools in Nigeria. Two schools do not have students in their final year and thus, were excluded from the study. All the final year dental students in the six dental schools were eligible to participate in the study. The co-investigators for this study administered the questionnaire prior to the commencement of a regular scheduled period of classroom instruction. All the students who were present in class were requested to fill the form after the objective and voluntary nature of the study had been explained. For students who were willing to participate in the study, their filled questionnaire was submitted to their respective class captains at the end of the classes. The class captains then returned the filled questionnaires to any of the co-investigators in their respective schools. All questionnaires were retrieved within a week of their administration.

## Questions and variables

Respondents were asked to indicate their gender (male or female) and age (at last birthday in years). The questionnaire requested information on respondents’ knowledge of preventive measures, attitude to preventive dentistry practice, and their own oral health behaviour.

### Knowledge of caries preventive measures

Respondents were asked to react to nine statements regarding various aspects of caries diagnosis and prevention on a five-point Likert scale ranging from ‘strongly agreed’ to ‘agreed’, ‘disagree’, ‘strongly disagree’ and ‘do not know’. The statements were: (i) Fluoridation of drinking water is an effective, safe, and efficient way to prevent dental caries (ii) Frequency of sugar consumption has a greater role in producing caries than the total amount of sugar (iii) Sealant is effective in the prevention of pit and fissure caries in newly erupted molars (iv) The probability of losing a restored tooth is greater when compared to losing a sound tooth (v) Rinsing teeth with a lower amount of water after tooth-brushing increases the effect of fluoride (vi) Examining a newly erupted tooth with a sharp explorer damages the enamel rods and makes the tooth vulnerable to caries (vii) A white or brown-spot lesion that is visible on a wet tooth surface has penetrated all the way through the enamel (viii) Using fluoride toothpaste is more important than the brushing *per se* for preventing caries (ix) Having dental problems can lead to general health problems. For each of the nine statements, respondents who indicated ‘strongly agreed’ and ‘agreed’ as options were graded as having responded correctly for the statement.

The responses were then scored from one to five with ‘strongly agreed’ scoring 5 and ‘do not know’ scoring 1. Where there were no responses, the score for 1 was allocated. Each respondent could therefore obtain a total minimum score of 9 and a total maximum score of 45. The mean scores for each respondent were calculated. This was used as the final knowledge score for each respondent. In order to dichotomise the variable, the median of the final scores served as cut-off point, with respondents scoring below the median categorised as having poor knowledge and all others comprising those with good knowledge. The median score for this sample was 4.

### Attitude to preventive dentistry practice

A seven-point semantic differential scale of eight qualities and their opposites was used to record the respondents' attitudes towards preventive dentistry. The qualities were: *Costly for the dentist/Not costly to the dentist, Not useful for the community/Useful for the community, Non-prestigious/Prestigious, Not effective/Effective, Non-essential/Essential, Unscientific/Scientific, Difficult/Simple, and Not Valuable/Valuable*. Scores were given to the responses (from one to seven, with the higher scores for the more favourable attitudes). Possible scores ranged from a minimum of 8 to a maximum of 56. Final attitude scores calculated and dichotomised as described above, with respondents scoring below the median categorised as having negative attitude and all others comprising those with positive attitude.

### Oral health behaviour

The respondents were requested to report the frequency with which they brush their teeth, use fluoridated toothpaste, floss and eat sugary snacks between main meals. These questions were used to determine self-care levels. These questions had four to seven alternatives. In order to define acceptable levels of each of the components, the following cut-off points were used: brushing more than once a day, using fluoridated toothpaste always or almost always, flossing at least once a day, and eating sugary snacks between main meals less frequently than once a day. Recommended oral self-care was defined as a composite score derived from indications of brushing teeth at least twice a day, use of fluoridated toothpaste, and consumption of sugary snacks between main meals less frequently than once a day [15,16].

The respondents were also asked to indicate the provider of their own dental check-ups (with the alternatives: *a dentist, a classmate*, *myself*, and *no need*) and the time of the last check-up (with the alternatives: *within the last 6 months, more than 6 months to one year ago, more than 1 to 2 years ago, more than 2 to 5 years ago, more than 5 years, never, do not remember*). Attending a dental check-up within the last year and provision of their own dental check-ups by a classmate and/ or a dentist was defined as preventive care use.

The questionnaire also requested information on the respondents’ cigarette smoking habits. The question had six alternatives. To dichotomize the variable, those who reported no present smoking will be considered as non-smokers.

## Inferential analysis

Chi-square test was used to test for significant differences between subgroups. Binary logistic regression models were fitted to the data to calculate odds ratios (OR) and confidence intervals (95% CI) for each of the four oral self-care measures. The independent variables for the model were gender, age, attitude towards preventive dentistry and knowledge of caries prevention scores. Age was dichotomised using the median age as the point of dichotomisation. The binary logistic regression model was used to calculate the association of the independent variables with dependent variables (tooth brushing more than once a day, intake of sugary snacks less than once a day, regular use of fluoride toothpaste, and use of dental floss every day or more). Association between the independent variables and recommended oral self-care was also assessed. STATA version 10 was used for data processing and statistical analysis.

Ethical clearance for the study was obtained from the Obafemi Awolowo University Teaching Hospitals Complex Health Research Ethics Committee.

## Results

There were only 223 students eligible for the study. Of these, 179 students filled the questionnaire giving a response rate of 80.3%. One hundred and seventy seven respondents indicated their gender in the questionnaire of which 106 (59.2%) males and 71 (39.7%) females. Two (1.1%) respondents did not indicate their sex. Also, 0nly 168 respondents gave their age. The age range of respondents was 21 years to 48 years and mean age of respondents was 27.2 years ± 3.2 years. These questionnaires with no data indicative of sex and age were excluded from the analysis where these variables were been analysed.

### Knowledge of caries preventive measures

More than 90% of the respondent agreed that (i) Fluoridation of drinking water is an effective, safe, and efficient way to prevent dental caries – 96.0% (ii) Frequency of sugar consumption has a greater role in producing caries than the total amount of sugar – 98.8% (iii) Sealant is effective in the prevention of pit and fissure caries in newly erupted molars – 92.7% and (iv) The probability of losing a restored tooth is greater when compared to losing a sound tooth – 92.7%. Majority (62.2%) of respondents did agreed that rinsing the teeth with a lower amount of water after tooth-brushing increases the effect of fluoride; 58.8% agreed that examining a newly erupted tooth with a sharp explorer damages the enamel rods and makes the tooth vulnerable to caries; 51.0% agreed that a white or brown-spot lesion that is visible on a wet tooth surface has penetrated all the way through the enamel; and 92.2% agreed that having dental problems can lead to general health problems. On the other hand, 53.7% of respondents disagreed or did not know that using fluoride toothpaste is more important than the brushing *per se* for preventing caries.

There was a statistically significant gender difference in the response ‘using fluoride toothpaste is more important than the brushing *per se* for preventing caries’: more males agreed with this statement (p<0.01). There were no age or gender differences observed with responses on any of the other statements.

### Oral health behaviour

Eighty five (48.0%) respondents brush once a day while 84 (47.5%) brush more than once a day. Up to 86 (48.6%) of respondents had never used a dental floss and only 13 (7.3%) of respondents used dental floss once a day or more. More females use dental floss (p=0.03). One hundred and sixty nine (95.4%) respondents used fluoridated toothpaste. Seventy seven (43.5%) respondents occasionally take sugar containing snacks in between meals, 47 (26.5%), 26 (14.7%) and 19 (10.7%) taken in-between once, twice or more than twice a day respectively. Most of the respondents (61.0%) conducted that last dental check up in the last six months while 12 (6.8%) had never conducted a dental check-up. Also, younger respondents had dentists or their classmate conduct their dental check-up for them (p=0.001). up to 162 (91.5%) of the study population had never smoked. Eleven (6.2%) has smoked but had quit smoking at the time of the survey.

### Oral self-care

Only 24.3% of respondents fulfilled the criteria for recommended oral self-care. No significant gender differences were observed. Older respondents were however, more likely to fulfill the criteria for recommended oral self-care (p<0.001). See Table 1. Those with good caries prevention knowledge reported less consumption of sugar in between meal (p=0.005). See Table 2. Respondents’ attitude had no impact on any of the oral health behaviours investigated in this study. See Table 2.

**Table 1:** Percentage of Nigerian dental students complying with various oral health behaviours according to their gender and age

**ROSC and its component**	*Gender		**P value**	*Age Group (years)		**P value**
	**Male**	**Female**		**22- 27**	**28 - 48**	
	**n = 106**	**n = 71**		**N =103**	**N =65**	
Tooth brushing more than once a day –R	50.0	43.7	0.71	46.6	46.2	0.96
Use of fluoridated toothpaste always –R	94.3	97.2	0.64	57.3	43.1	0.07
Between meal sugar consumption less than once daily – R	55.7	35.2	0.24	95.1	87.7	0.50
Dental flossing at least once a day	4.7	11.3	**0.03	4.9	4.6	0.94
Dental checkup within past year	47.2	81.7	0.13	35.0	33.8	0.93
Dental check up by dentists or classmate	77.4	66.2	0.40	63.1	33.8	**0.001
Non smoking	97.2	98.6	0.80	82.5	69.2	0.15
Recommended oral self-care - 3xR	29.2	16.9	0.37	14.6	43.1	**0.00

*only 177 and 168 of the 179 respondents indicated their gender and age respectively.

**statistically significant difference R – the elements included in the recommended oral self-care package.

**Table 2:** Percentage of Nigerian dental students complying with various oral health behaviours according to their level of knowledge of preventive dental care

**Oral health behaviour**	**Total**	**Knowledge**		**P value**	**Attitude**		**P value**
		**Good**	**Poor**		**Positive**	**Negative**	
		**n=85**	**n=94**		**n=97**	**n=82**	
Tooth brushing more than once a day	85	45	40	0.69	49.5	45.1	0.56
Use of fluoridated toothpaste always	171	89	82	0.42	94.8	91.5	0.63
Between meal sugar consumption less than once daily	85	53	32	*0.005	47.4	47.6	0.99
Dental flossing at least once a day	13	6	7	0.70	10.3	3.7	0.09
Dental check up within past year	119	59	60	0.49	66.0	67.0	0.88
Dental check-up by dentists or classmates	139	67	72	0.11	77.3	80.4	0.40
Non smoking	175	90	85	0.96	96.9	98.7	0.40
Recommended oral self care	44	28	16	0.14	24.7	24.3	0.95

### Determinants for complying with optimal oral health behaviours among senior dental students

In the binary logistic regression models (Table 3), good knowledge of preventive dental care measures and respondents who were older were more likely to take sugary snacks less than once a day. Good knowledge of preventive dental care was associated with one fold increase in tendency to brush the teeth more than once a day, and a five-fold increase in the use of recommended oral self-care. Also, a positive attitude to preventive dentistry was associated with over a 3 fold increase in the tendency to use of dental floss daily or more than once a day. These findings were however, not statistically significant.

**Table 3:** Association between oral health behaviour component and selected variables among Nigerian dental students (N=168)

**Variables**	**SE**	**OR**	**95% CI**	**P value**
**Toothbrushing more than once a day**				
Female Gender	0.14	0.92	0.68 -1.24	0.57
Age (22 to 27 years)	1.12	2.2	0.84 – 5.96	0.11
Good knowledge of preventive dental care	0.32	1.0	0.55 -1.90	0.94
Positive attitude to preventive dentistry	0.38	1.2	0.64 -2.21	0.60
**Sugary snacks less than once a day**				
Female Gender	0.15	0.8	0.59 -1.18	0.31
Age (22 to 27 years)	1.59	3.0	1.05 – 8.49	*0.04
Good knowledge of preventive dental care	0.78	2.4	1.26 -4.54	*0.008
Positive attitude to preventive dentistry	0.28	0.8	0.45 -1.61	0.61
**Regular use of fluoride toothpaste**				
Female Gender	1.98	2.3	0.44 -12.27	0.32
Age (22 to 27 years)	1.14	1.0	0.12 – 9.11	0.98
Good knowledge of preventive dental care	1.82	2.4	0.52 – 10.71	0.27
Positive attitude to preventive dentistry	0.41	0.5	0.12 -2.41	0.41
**Use of dental floss every day or more**				
Female Gender	0.23	1.0	0.66 – 1.58	0.93
Age (22 to 27 years)	1.10	1.3	0.27 -6.73	0.72
Good knowledge of preventive dental care	0.36	0.6	0.19 – 1.93	0.39
Positive attitude to preventive dentistry	2.36	3.4	0.88 – 13.24	0.08
**Recommended oral self care**				
Female Gender	0.83	1.2	0.32 – 4.60	0.77
Age (22 to 27 years)	0.58	0.5	0.05 - 4.97	0.55
Good knowledge of preventive dental care	6.20	5.4	0.58 -51.12	0.14
Positive attitude to preventive dentistry	0.54	0.6	0.09 – 3.70	0.56

*SE* Standard Error, *OR* Odds ratio.

## Discussion

There is limited information available about dentists’ oral health behaviour including their preventive oral health practices. The importance of the use of fluoridated toothpaste for the prevention of caries has been well studied. Its use has been associated with the decline in caries incidence observed in many developed countries. For this study however, just about 46% agreed that fluoridated toothpaste is more important than tooth brushing technique in caries prevention. This may be a reflection that the current dental curriculum may be placing a lot more emphasis on the traditional preventive measures which focus on oral hygiene aspects of caries prevention. This observation had been made earlier in Mongolia, Korea and Canada [5,17,18]. It would therefore be needful that practicing dental professionals are mobilised to update their knowledge as new oral health management strategies are identified. It will also be needful to review the dental education curriculum with efforts targeted at enabling dental students’ build their knowledge and attitude based on current updates. Knowledge and attitude is important as these serves as framework for practice [7].

The study also observed that there was significant gender disparity in the agreement on that fluoridated toothpaste was more important than tooth brushing technique in caries prevention. The reason for this observed gender disparity cannot be readily explained. The possible role of cultural differences in oral health attitudes and behaviours due in part to the role placed by socio-environmental factors in oral health-related behaviours may be a key consideration [19,20]. Culture may however, not be able to explain this specific gender difference observed with respect to agreement on the statement that fluoridated toothpaste been more important than tooth brushing technique in caries prevention.

The frequency of twice daily brushing among dental students in Nigeria is very low (47.5%) compared to their counterparts in Mongolia (81%) [16], France (78%) [21], Iran (57%) [15] and Australia (80%) [22]. It is even less than what has been reported among the general public in Germany, Japan, New Zealand, the USA, and Poland [23], the UK [24] and Finland [25,26]. The effects of various dental curricula on these differences have also been emphasised [5,17,18]. Preventive dentistry is an institutionalised course in all dental schools in Nigeria. The impact of specific training on preventive dentistry is reflected in the high number of respondents who demonstrated good knowledge about caries preventive practices: more than 90% agreed about the importance of fluoridated toothpaste, frequency of sugar consumption, and the use of fissure sealants as effective for caries prevention. There however, appears to be a gap in the oral health practice of dental students and what is taught in the oral health curriculum. Not only does the preventive dentistry dental school curriculum emphasise twice daily brushing, the national oral health programme currently run in the country also publicly advertise and promote twice daily brushing. What we may be observing is a slow shift in oral health behaviour. Such possible shift in attitude and behaviour amongst dental students has been observed in Australia [22]. It may be important to conduct a study to identify if there is truly a slow but evolving change in the frequency of tooth brushing among dental students in Nigeria. In the absence of such evolving change, measures may need to be taken to promote twice daily tooth brushing by dental students in Nigeria.

While over 95% of the students use fluoridated toothpastes - higher than what is observed in Mongolia [16] and Iran [15]; more than half of the students (53.5%) still consume sugary snacks in between meals daily - a figure that is similar to what is observed in Iran [15] and Mongolia [16] but lower than what is reported in Australia [22].

Overall, just about 23% of respondents practice recommended oral self-care habits. While significant gender differences existed in the use of recommended oral self-care in Iran [15] and Jordan [12,27] in contrast to Mongolia [16] and Spain [3], none of these studies have reported age differences in the practice of self-oral care. This study observed significant age differences in the practice of recommended oral self-care with older students being more inclined to adhere to recommended oral self-care. The logistic regression analysis however showed that although older students were more inclined to practice recommended oral self-care, the observed age difference was not significant.

Knowledge and attitude are considered factors that predispose to action [7,14,18]. In the present study, good knowledge of preventive dental care was associated with better compliance with recommended oral self-care. On the other hand, attitude toward preventive dentistry practice was not significantly associated with any oral health behaviour components in the study. This observation may well support the notion that knowledge impacts on the dental student’s dental health-related behaviour. It is important to interpret with caution the observation on attitude since the assessment was their attitude towards practicing preventive dentistry as a profession and less so as a routine daily preventive oral health care practice.

There were only 8.4% of the respondents who ever smoked cigarette. Of these, 73.3% had quit at the time of the study. Smoking is reported to be rare among dentists, with dentists reported to have the lowest rate of smoking among all health professionals [28] though contrary findings were reported among dentists from Italy [29] and Jordan [30]. The low prevalence of smoking among the dental students may also be a reflection of the socio-cultural norms: Nigeria is a highly religious country where smoking and the use of alcoholic beverages is highly frowned upon.

All study participants were dental students thereby reducing the probability of biases related to misconceptions and errors in interpretation of concepts which exist in studies using self-administered questionnaires with lay populations [31,32]. Despite this, the tendency towards socially desirable response cannot be completely excluded especially when using a self-assessment tool [33]. Self-assessment questionnaires however, remain a quick, practical, and economical way of data collection especially among adult populations [34]. In order to get accurate responses, an effort was made to provide a wide range of possible answers.

## Conclusion

In conclusion, while there was an appreciably high level of good knowledge of preventive dental care amongst these Nigerian students, this does not seem to be an equally appreciable impact on their oral health behaviour with less than a third of them practicing recommended self-care measures. Also, dental students’ background variables such as gender, age and knowledge of caries prevention do inform differences in their preventive dental care. Future researches should help identify how and why such background variables are significant determinants of oral health behaviour in dental students despite their professional training.

## Competing interests

The authors declare that they have no competing interest.

## Authors’ contributions

MOF conceptualised the study, was involved with the design, data compilation, analysis and writing up of the study outcome. MRK was involved with the study design, data analysis, writing up of the manuscript. NF was involved with the study design, data collection and writing of the manuscript. BP was involved with the study design, data collection and writing of the manuscript. OOS was involved with the study design, data collection and writing of the manuscript. TOL was involved with the study design, data collection and writing of the manuscript. AOE was involved with the study design, data collection and writing of the manuscript. OO was involved with the study design, data collection and writing of the manuscript. All authors read and approved the final manuscript.

## Acknowledgement

The authors acknowledge the contributions made by Dr Antony Osaguona with respect to data collection in his institution.

## Pre-publication history

The pre-publication history for this paper can be accessed here:

http://www.biomedcentral.com/1472-6831/13/28/prepub
